# Supplementary material for: Suppressing dipolar relaxation in thin layers of dysprosium atoms
Source: Nat Commun. 2024 Apr 26;15:3566. doi: 10.1038/s41467-024-47260-1 (PMC11052996; doi:10.1038/s41467-024-47260-1)
Supplement: Supplementary file 1 — Supplementary Information [file 41467_2024_47260_MOESM1_ESM.pdf]

Suppressing dipolar relaxation in thin layers of dysprosium atoms

Pierre Barral[1†], Michael Cantara[1†], Li Du[1†], William Lunden[1], Julius de Hond[1], Alan O. Jamison[1] and Wolfgang Ketterle[1]

[1]Research Laboratory of Electronics, MIT-Harvard Center for Ultracold Atoms, and Department of Physics, Massachusetts Institute of Technology, Cambridge, Massachusetts 02139, USA.

[†]These authors contributed equally to this work.

Supplementary Information

Supplementary Note 1: Introduction

We present here our theoretical model to compute the expected wave functions shown in Fig. 1 and rates shown in Fig. 2 and 3 of the main text. The calculations use the formalism of Fermi's golden rule. For completeness, we also derive and discuss the Born approximation previously sketched in [1] and used extensively in [2]. We comment on their equivalence and then discuss the pure-2D limit. We finally provide some comments on dimensionality and magnetic field scaling.

Both Fermi's golden rule and the Born approximation are perturbative approaches to quantum scattering. The former decomposes incoming and outgoing waves into many different channels, reducing the calculation effectively to 1D (i.e. the radial coordinate). The latter computes the perturbative impact of the dipolar relaxation Hamiltonian on an incoming plane wave, leaving the calculation in 2D. Both results are equivalent. It is easier to generalize Fermi's golden rule for our approach, which uses initial wave functions modified by the dipolar elastic potential. The Born approximation will be presented only

for un-modified plane waves, whereas Fermi's golden rule formalism will be applied to both modified and un-modified wave functions.

Dipolar units

For the following derivations, it is convenient to use dipolar units, indicated with a tilde. Note that we use the dipolar length's definition $a_{\mathrm{dd}} = \frac{\mu_0}{4\pi}\frac{\mu(10\mu_B)^2}{\hbar^2}$ used broadly for 2-body collisions. In a many-body physics context, the alternative definition $a_{\mathrm{dd}} = \frac{\mu_0}{4\pi}\frac{2\mu(10\mu_B)^2}{3\hbar^2}$ is more common. Dimensionless lengths are set with the dipolar length, such that $\tilde{a}_z = a_z/a_{\mathrm{dd}}$, wave functions become $\tilde{\phi} = a_{\mathrm{dd}}^{1/2}\phi$, momenta become $\tilde{k}_i = k_i a_{\mathrm{dd}}$ and energies are measured in units of dipolar energy $E_{\mathrm{dd}} = \frac{\hbar^2}{2\mu a_{\mathrm{dd}}^2}$, so $\tilde{V}_{\mathrm{dd}} = V_{\mathrm{dd}}/E_{\mathrm{dd}}$. The equation (4) becomes

$$\hat{V}_{\mathrm{dd}}\left|j_0\right\rangle = \frac{2E_{\mathrm{dd}}}{\tilde{r}^3}\left[\left(1-3\bar{z}^2\right)\left|j_0\right\rangle - \frac{3\bar{z}\bar{r}_+}{J^{1/2}}\left|j_1\right\rangle - \frac{3\bar{r}_+^2}{2J}\left|j_2\right\rangle\right] \tag{SI-1}$$

and equation (6) for an arbitrary harmonic oscillator channel n becomes

$$\left(-\frac{\mathrm{d}^2}{\mathrm{d}\tilde{\rho}^2} + \frac{m^2 - 1/4}{\tilde{\rho}^2} + \langle n|\,\tilde{V}_{\mathrm{dd},\,0}\,|n\rangle\right)\tilde{\phi}_{n,m} = \tilde{k}_i^2\tilde{\phi}_{n,m}. \tag{SI-2}$$

The normalization condition reads: $\int_0^{\tilde{L}} \mathrm{d}\tilde{\rho}\,\tilde{\phi}_{n,m}(\tilde{\rho})^2 = 1$ for a cylinder of radius $L = \tilde{L}a_{\mathrm{dd}}$. Our model accounts for the modification of both incoming and outgoing wave functions by the dipolar interaction. The free radial wave function solution with momentum k is $\tilde{\phi}_{n,m}^{(\mathrm{free})}(\tilde{\rho}) = \sqrt{\frac{\pi\tilde{k}\tilde{\rho}}{\tilde{L}}}J_m(\tilde{k}\tilde{\rho})$, which does not depend on n.

Supplementary Note 2: Fermi's golden rule derivation

Here we derive the expression for the 3D loss rate coefficient for the channel $|j_0\rangle \to |j_f\rangle, |0\rangle \to |n_f\rangle$:

$$\beta_{3\mathrm{D}}^{j_f,n_f} = \frac{4\sqrt{\pi}}{\tilde{k}_i\tilde{k}_f}\tilde{a}_z\frac{E_{\mathrm{dd}}}{\hbar}a_{\mathrm{dd}}^3\left|\tilde{L}\int \mathrm{d}\tilde{z}\int_0^{\tilde{L}}\mathrm{d}\tilde{\rho}\,\tilde{\phi}_{n_f,j_f}(\tilde{\rho})\tilde{\chi}_{n_f}(\tilde{z})\tilde{V}_{\mathrm{dd},j_f}(\tilde{\rho},\tilde{z})\tilde{\chi}_0(\tilde{z})\tilde{\phi}_0(\tilde{\rho})\right|^2.$$

$\tilde{\chi}_n$ are the harmonic oscillator wave functions in the z direction. $\tilde{a}_z$ is the harmonic oscillator length in units of the dipolar length.

Fermi's golden rule

Fermi's golden rule conveniently expresses the decay rate from an initial state to a continuum having a certain density of states. It is natural to compute Fermi's golden rule with free incoming and outgoing plane waves, which we do at first. We then expand it into cylindrical waves, which we finally substitute for their shielded version solutions of equation (SI-2).

The starting point is using equation (2) to compute the decay rate $\Gamma_{\mathrm{plane}}^{j_f,n_f}$ of a pair of polarized atoms coming in a plane wave in the ground state of the harmonic oscillator, and outgoing in another asymptotic plane wave in the oscillator state n_f with a spin state $|j_f\rangle$. The particles are assumed to be contained in a cylinder of length L and by a harmonic oscillator potential in the z direction.

$$\hbar\Gamma_{\mathrm{plane}}^{j_f,n_f} = 2\pi\left|\left\langle\Psi_{j_f,n_f}\right|\hat{V}_{\mathrm{dd}}\left|\Psi_{j_0,0}\right\rangle\right|^2\rho_p(E_f),$$

with the total wave function $|\Psi_{j,n}\rangle = |\boldsymbol{k}\rangle \otimes |n\rangle \otimes |j\rangle$ and

$$\langle\boldsymbol{\rho}|\boldsymbol{k}\rangle = \frac{e^{i\boldsymbol{k}\cdot\boldsymbol{\rho}}}{\sqrt{\pi L^2}}$$

$$\langle z|n\rangle = \frac{1}{\sqrt{2^n n!}}\left(\frac{1}{\pi a_z^2}\right)^{1/4}H_n(z/a_z)e^{-\frac{z^2}{2a_z^2}} = \chi_n(z)$$

Density of states

In a two-dimensional box, the volumic density of states is $\frac{\mu}{2\pi\hbar^2}$. In a particular direction of angle $\mathrm{d}\theta_k$, the density of states is $\rho_p(k_f) = \frac{\mu L^2}{4\pi\hbar^2}\,\mathrm{d}\theta_k$.

Plane wave expansion

To fully use the symmetries of the dipolar potential, one can expand the plane wave into spherical waves:

$$|\boldsymbol{k}\rangle = \sqrt{\frac{2}{\pi k_i L}} \sum_{m=-\infty}^{+\infty} i^m e^{-im\theta_k} |k, m\rangle, \tag{SI-3}$$

which have the following position representation:

$$\langle \boldsymbol{\rho} | k, m\rangle = \frac{e^{im\theta}}{\sqrt{2\pi}} \frac{\phi_m(\rho)}{\sqrt{\rho}}. \tag{SI-4}$$

Summing over all possible outgoing directions, the rate is

$$\hbar\Gamma^{j_f, n_f}_{\text{plane}} = 2\pi \int \mathrm{d}\theta_f \left| \sum_{m_i=-\infty}^{+\infty} \sum_{m_f=-\infty}^{+\infty} i^{m_i - m_f} e^{-im_i\theta_i + im_f\theta_f} \right.$$
$$\left. \langle k_f, m_f| \langle n_f| \langle j_f| \hat{V}_{\text{dd}} |j_0\rangle |0\rangle |k_i, m_i\rangle \right|^2 \frac{2}{\pi k_i} \frac{2}{\pi k_f} \frac{\mu}{4\pi\hbar^2}. \tag{SI-5}$$

Dipolar interaction

To simplify the problem we can look at the selection rules of the dipolar potential. The equation (3) can be written

$$\begin{aligned}
\hat{V}_{\text{dd}} &= \frac{\mu_0}{4\pi}(g_J\mu_B)^2 \frac{\hat{\boldsymbol{J}}_{\boldsymbol{1}} \cdot \hat{\boldsymbol{J}}_{\boldsymbol{2}} - 3(\hat{\boldsymbol{J}}_{\boldsymbol{1}} \cdot \boldsymbol{u_r})(\hat{\boldsymbol{J}}_{\boldsymbol{2}} \cdot \boldsymbol{u_r})}{r^3} \\
&= \frac{\mu_0}{4\pi}(g_J\mu_B)^2 \frac{1}{r^3} \{ \hat{J}_{1z}\hat{J}_{2z} \left(1 - 3\bar{z}^2\right) \\
&\quad + \frac{1}{2}\left(\hat{J}_{1+}\hat{J}_{2-} + \hat{J}_{1-}\hat{J}_{2+}\right) \\
&\quad - \frac{3}{2}\bar{z}\left[\hat{J}_{1z}\left(\hat{J}_{2+}\bar{r}_- + \hat{J}_{2-}\bar{r}_+\right) + \hat{J}_{2z}\left(\hat{J}_{1+}\bar{r}_- + \hat{J}_{1-}\bar{r}_+\right)\right] \\
&\quad - \frac{3}{4}\left(\hat{J}_{1+}\bar{r}_- + \hat{J}_{1-}\bar{r}_+\right)\left(\hat{J}_{2+}\bar{r}_- + \hat{J}_{2-}\bar{r}_+\right) \}
\end{aligned}$$

which gives equation (4)

$$\hat{V}_{\text{dd}} |j_0\rangle = \tfrac{2E_{\text{dd}}}{\bar{r}^3} \left[\left(1 - 3\bar{z}^2\right)|j_0\rangle - \tfrac{3\bar{z}\bar{r}_+}{J^{1/2}}|j_1\rangle - \tfrac{3\bar{r}_+^2}{2J}|j_2\rangle\right]. \tag{SI-6}$$
$$= V_{\text{dd},0}|j_0\rangle + V_{\text{dd},1}|j_1\rangle + V_{\text{dd},2}|j_2\rangle \tag{SI-7}$$

with $\bar{r}_+ = \frac{\rho}{r}e^{i\theta}$ and $\bar{z} = z/r$. Hence $V_{\text{dd},0}$ is independent of θ, $V_{\text{dd},1}$ proportional to $e^{i\theta}$ and $V_{\text{dd},2}$ to $e^{2i\theta}$. The $e^{im\theta}$ in equation (SI-4) makes the matrix elements $\langle k_f, m_f| \hat{V}_{\text{dd},j_f} |k_i, m_i\rangle$ of the sum (SI-5) non-zero only if

$m_f = m_i + j_f$. Furthermore, as $\bar{z}$ is anti-symmetric, and both χ_0 and $\bar{r}_+$ are symmetric, $V_{\mathrm{dd},1}$ can only promote to odd n_f states and $V_{\mathrm{dd},2}$ to even ones. This gives

$$\hbar\Gamma_{\mathrm{plane}}^{j_f,n_f} = \frac{2\mu}{\pi^2 k_i k_f \hbar^2} \int \mathrm{d}\theta_f \left| \sum_{m_i=-\infty}^{+\infty} e^{i m_i \theta_f} \langle k_f, m_i + j_f | \langle n_f | V_{\mathrm{dd},j_f} |0\rangle |k_i, m_i\rangle \right|^2 . \tag{SI-8}$$

Symmetrization

Since the atoms are bosons, the wave functions need to be symmetrized. All the spin states are already symmetrized. Each incoming and outgoing $|\boldsymbol{k}\rangle$ state from (SI-3) becomes $\frac{|\boldsymbol{k}\rangle + |-\boldsymbol{k}\rangle}{\sqrt{2}}$. It transforms the sum (SI-8) by multiplying the incoming and outgoing terms by $\sqrt{2}$ each and summing on even m_i for bosons and odd for fermions. The density of states of the outgoing channels is divided by 2 to avoid double counting. Furthermore, due to the independence of the braket on θ_f, the only terms in the sum giving a non-zero contribution after integrating over θ_f are the terms diagonal in m_i coming from the modulus. This gives:

$$\hbar\Gamma_{\mathrm{sym}}^{j_f,n_f} = \frac{8\mu}{\pi k_i k_f \hbar^2} \sum_{\text{even } m_i} \left| \langle k_f, m_i + j_f | \langle n_f | V_{\mathrm{dd},j_f} |0\rangle |k_i, m_i\rangle \right|^2 . \tag{SI-9}$$

s-wave scattering

Given our parameter range, we only keep the $m_i = 0$ channel, i.e. the s-wave channel. This reflects that the relevant range of the dipolar potential is much smaller than the incoming De Broglie wavelength. It comes from the fact that $k_f \gg k_i$ for most of the magnetic fields (see in the "Comparing Born approximation and Fermi's golden rule" paragraph of the Supplementary Information for a discussion when the magnetic energy is comparable to the temperature). The outgoing wave function starts to oscillate at a distance $\sim |m_f|/k_f \sim 1/k_f$ which cuts out the integration at this distance (see Fig. S4). Before that point, the free wave function $\phi_m(\rho)$ rises like a Bessel function in $\sqrt{\rho}(k\rho)^{|m|}$, and the dipolar potential goes as $1/\rho^3$. The integral goes then like $\int_0^{|m_f|/k_f} \rho\, \mathrm{d}\rho (k_f\rho)^{|m_f|} \frac{1}{\rho^3} (k_i\rho)^{|m_i|} \propto k_f^{|m_f|} k_i^{|m_i|} \frac{k_f^{-|m_i|-|m_f|+1}}{|m_i|+|m_f|-2} \propto k_f \left(\frac{k_i}{k_f}\right)^{|m_i|} \propto \sqrt{B}\left(\frac{k_B T}{B}\right)^{|m_i|/2}$, such that all the terms for which $m_i \neq 0$ are greatly suppressed as soon as the magnetic field energy is greater than the temperature (1μK, which is about 10 mG). Therefore

$$\hbar\Gamma_{\mathrm{sym}}^{j_f,n_f} = \frac{8\mu}{\pi k_i k_f \hbar^2} \left| \langle k_f, m_f = j_f | \langle n_f | V_{\mathrm{dd},j_f} |0\rangle |k_i, 0\rangle \right|^2 .$$

Wave function substitution

We have expanded the plane wave in equation (SI-3) into cylindrical wave functions, solutions of the Schrödinger equation for a free particle, i.e. equation (SI-2) without the dipolar interaction term. We now replace these free cylindrical wave functions ϕ_m by the solutions of the full Schrödinger equation with the dipolar interaction term $\phi_{n,m}$, which now depend on the harmonic oscillator channel n. The initial state is now the shielded wave function, and Fermi's golden rule describes its dipolar decay. This substitution is valid as the plane wave expansion (SI-3) still holds at large distance since the dipolar potential in $1/\rho^3$ decays faster than the centrifugal $1/\rho^2$ potential.

Calculation of $\beta_{2\mathrm{D}}$

The rate Γ_{sym} is the probability per unit of time of the two bosons decaying when placed in a harmonic oscillator state $n = 0$ in a cylindrical box of radius L. We are interested in the decay rate $\beta_{2\mathrm{D}}$ defined through $\frac{\mathrm{d}n_{2\mathrm{D}}}{\mathrm{d}t} = -\beta_{2\mathrm{D}} n_{2\mathrm{D}}^2$, which for a homogeneous gas gets integrated into $\frac{\mathrm{d}N}{\mathrm{d}t} = -\beta_{2D}\frac{N^2}{\pi L^2}$. For N atoms homogeneously spread in the area, the differential equation sums the decay rate on all the possible pair combinations $N(N-1)/2$. For each event two atoms get lost, so $\frac{\mathrm{d}N}{\mathrm{d}t} = -2\Gamma\frac{N(N-1)}{2} \sim -\Gamma N^2$ for large N. It gives $\beta_{2\mathrm{D}} = \pi L^2 \Gamma$:

$$\beta_{2\mathrm{D}}^{j_f,n_f} = \frac{8\mu}{k_i k_f \hbar^3}\left| L\int_{-\infty}^{+\infty} \mathrm{d}z \int_0^L \mathrm{d}\rho \phi_{n_f,j_f}(\rho)\chi_{n_f}(z)V_{\mathrm{dd},\,j_f}(\rho,z)\chi_0(z)\phi_0(\rho)\right|^2 .$$

The $\phi_{n,j}$ wave functions are either free wave functions like the light blue curve in Fig. 1b or modified wave functions through equation (6) such as the blue and navy curves on the same figure.

Calculation of $\beta_{3\mathrm{D}}$

The final equation for $\beta_{3\mathrm{D}}$ follows from equation (12), so

$$\beta_{3\mathrm{D}}^{j_f,n_f} = \frac{4\sqrt{\pi}}{\tilde{k}_i\tilde{k}_f}\tilde{a}_z\frac{E_{\mathrm{dd}}}{\hbar}a_{\mathrm{dd}}^3\left|\tilde{L}\int\int_0^{\tilde{L}} \mathrm{d}\tilde{z}\,\mathrm{d}\tilde{\rho}\,\tilde{\phi}_{n_f,j_f}(\tilde{\rho})\tilde{\chi}_{n_f}(\tilde{z})\tilde{V}_{\mathrm{dd},j_f}(\tilde{\rho},\tilde{z})\tilde{\chi}_0(\tilde{z})\tilde{\phi}_0(\tilde{\rho})\right|^2 \tag{SI-10}$$

Note that the prefactor $E_{\mathrm{dd}}a_{\mathrm{dd}}^3/\hbar$ is useful to check the units but inconvenient to look at the scaling with the dipolar interaction. When the wave functions are not modified by the dipole-dipole potential they read: $\tilde{\phi}_{n,m}(\tilde{\rho}) = \sqrt{\frac{\pi\tilde{k}\tilde{\rho}}{\tilde{L}}}J_m(\tilde{k}\tilde{\rho})$

and $\tilde{\chi}_n(\tilde{z}) = \frac{1}{\sqrt{2^n n!}}\left(\frac{1}{\pi\tilde{a}_z^2}\right)^{1/4} H_n(\tilde{z}/\tilde{a}_z)e^{-\frac{\tilde{z}^2}{2\tilde{a}_z^2}}$, which gives for the 2D rate:

$$\beta_{\rm 2D}^{\rm free} = \frac{9}{2\pi J^2}\frac{\hbar}{\mu}a_{\rm dd}^2\left|\int_{-\infty}^{+\infty}\int_0^L {\rm d}\xi\, 2\pi\rho\, {\rm d}\rho\ J_{j_f}(k_f\rho)\frac{\rho^2}{(\rho^2+\xi^2\tilde{a}_z^2)^{3/2}}J_0(k_i\rho)\right.$$
$$\left.\frac{1}{\sqrt{2^{n_f}n_f!}}H_{n_f}(\xi)e^{-\xi^2}\right|^2$$

which explicitly shows the $a_{\rm dd}^2 \propto (10\mu_B)^4$ scaling.

Total decay rate

The total rate is then the sum over all channels:

$$\beta_{\rm 3D} = \sum_{j_f,n_f}\beta_{\rm 3D}^{j_f,n_f}.$$

Momentum averaging

The rate obtained depends on the incoming momentum $\tilde{k}_i$. The gas being thermal with many occupied states in the transverse direction, we integrate over momentum to obtain the average decay rate

$$\bar{\beta}_{\rm 3D} = \frac{1}{\pi\kappa^2}\int_0^\infty 2\pi k\beta_{\rm 3D}(k)e^{-k^2/\kappa^2}\,{\rm d}k$$

with $\kappa = \sqrt{k_BT/E_{\rm dd}}$. The results presented in Fig. 2b-c are the average momentum rates. But since this is rather computationally heavy and blurs the channel opening, we only present results computed at the mean momentum $\overline{\tilde{k}_i} = \kappa\sqrt{\pi}/2$ in the rest of the paper. For instance, the incoming energy of the wave functions in Fig. 1a-b is $E_i = E_{\rm dd}\kappa^2\pi/4$.

Supplementary Note 3: Born approximation derivation

Definitions

The Born approximation is the standard way to describe scattering by a potential. The method in 2D has been described in [1], but it is lacking of an explicit final formula, which we would like to present here. We look for eigenstates of the full Hamiltonian:

$$(E - \hat{H}_0) |\Psi\rangle = \hat{V}_{\mathrm{dd}} |\Psi\rangle \tag{SI-11}$$

with

$$\hat{H}_0 = H_{2D} + H_n + \hat{H}_B = -\frac{\hbar^2}{2\mu}\nabla^2_{2D} + \hbar\omega_z\left(n + \frac{1}{2}\right) + \mu_B g_J B_z\left(\hat{J}_{z,1} + \hat{J}_{z,2}\right) \tag{SI-12}$$

and $\hat{V}_{\mathrm{dd}}$ defined through equation (SI-6). We define the Green operator $\hat{G}_0$ as $(E - \hat{H}_0 \pm i\eta)\hat{G}_0 = \mathbb{1}$ and will eventually take the limit $\eta \to 0$. We omit the surface normalization coefficient in the wave function such that: $\langle \boldsymbol{\rho}|\boldsymbol{k}\rangle = e^{i\boldsymbol{k}\cdot\boldsymbol{\rho}}$; $\langle \boldsymbol{\rho}|\boldsymbol{\rho}'\rangle = \delta_{2D}(\boldsymbol{\rho} - \boldsymbol{\rho}')$ and $\langle \boldsymbol{k}|\boldsymbol{k}'\rangle = (2\pi)^2\delta_{2D}(\boldsymbol{k} - \boldsymbol{k}')$. It follows that:

$$\mathbb{1}_{\mathrm{2D}} = \frac{1}{(2\pi)^2}\int \mathrm{d}^2\boldsymbol{k}\,|\boldsymbol{k}\rangle\langle\boldsymbol{k}| = \int \mathrm{d}^2\boldsymbol{\rho}\,|\boldsymbol{\rho}\rangle\langle\boldsymbol{\rho}|$$

$$\Psi(\boldsymbol{\rho}) = \langle\boldsymbol{\rho}|\Psi\rangle = \frac{1}{(2\pi)^2}\int \mathrm{d}^2\boldsymbol{k}\,\langle\boldsymbol{\rho}|\,|\boldsymbol{k}\rangle\langle\boldsymbol{k}|\,|\Psi\rangle = \frac{1}{(2\pi)^2}\int \mathrm{d}^2\boldsymbol{k} e^{i\boldsymbol{k}\cdot\boldsymbol{\rho}}\psi(\boldsymbol{k})$$

$$\psi(\boldsymbol{k}) = \langle\boldsymbol{k}|\Psi\rangle = \int \mathrm{d}^2\boldsymbol{\rho} e^{-i\boldsymbol{k}\cdot\boldsymbol{\rho}}\Psi(\boldsymbol{\rho})$$

.

Born approximation

Following the definitions, $|\Psi\rangle = \hat{G}_0\hat{V}_{\mathrm{dd}}|\Psi\rangle$ is one solution of the eigenvalue equation (SI-11). If furthermore we have a solution $|\Psi_0\rangle$ to the equation $(E - \hat{H}_0)|\Psi_0\rangle = 0$ then $|\Psi\rangle = |\Psi_0\rangle + \hat{G}_0\hat{V}_{\mathrm{dd}}|\Psi\rangle$ is also a solution. The first Born approximation consists in computing the first-order part of the solution:

$$|\Psi\rangle = |\Psi_0\rangle + \hat{G}_0\hat{V}_{\mathrm{dd}}|\Psi_0\rangle \tag{SI-13}$$

Let us take $|\Psi_0\rangle = |\boldsymbol{k_i}\rangle \otimes |n = 0\rangle \otimes |j_0\rangle$ which has the energy: $E = \frac{\hbar^2 k_i^2}{2\mu} + \frac{1}{2}\hbar\omega_z + 16\mu_B g_J B_z$. Note that we will later symmetrize the incoming state by replacing $|\boldsymbol{k_i}\rangle$ by $\frac{|\boldsymbol{k_i}\rangle + |-\boldsymbol{k_i}\rangle}{\sqrt{2}}$, so it is good to keep track of the $e^{i\boldsymbol{k_i}\cdot\boldsymbol{\rho}}$ terms that will eventually become $\frac{e^{i\boldsymbol{k_i}\cdot\boldsymbol{\rho}} + e^{-i\boldsymbol{k_i}\cdot\boldsymbol{\rho}}}{\sqrt{2}}$.

Calculation of $\psi_{n_f,j_f}(\boldsymbol{k})$

We decompose the problem into channels (n_f, j_f). We eventually want the position representation of the scattered wave function but the Green operator has a simpler expression in momentum space. When projecting $|\Psi\rangle$ on $\langle j_f| \langle n_f| \langle \boldsymbol{k}|$ the first term in equation (SI-13) disappears for $(n_f, j_f) \neq (n_i = 0, j_0)$. The matrix element to compute is thus

$$\psi_{n_f,j_f}(\boldsymbol{k}) = \langle j_f| \langle n_f| \langle \boldsymbol{k}|\hat{G}_0\hat{V}_{\rm dd}|\boldsymbol{k_i}\rangle |0\rangle |j_0\rangle$$

When acted on the left side, the Green operator passes through the states which are the eigenstates of $\hat{H}_0$ from equation (SI-12), giving

$$\psi_{n_f,j_f}(\boldsymbol{k}) = \frac{1}{E - \frac{\hbar^2 k^2}{2\mu} - \hbar\omega_z\left(n_f + \frac{1}{2}\right) - \mu_B g_J B_z(16-\Delta m) \pm i\eta} \langle j_f| \langle n_f| \langle \boldsymbol{k}|\hat{V}_{\rm dd}|\boldsymbol{k_i}\rangle |0\rangle |j_0\rangle \tag{SI-14}$$

with $\Delta m = 1, 2$ for channels j_1, j_2 respectively. By setting:

$$\frac{\hbar^2 k_f^2}{2\mu} = \frac{\hbar^2 k_i^2}{2\mu} - \hbar\omega_z \Delta n + \mu_B g_J B_z \Delta m$$

with $\Delta n = n_f - n_i$, the denominator can be simplified to $\frac{\hbar^2}{2\mu}\left(k_f^2 - k^2 \pm i\eta\right)$. We introduce the identity $\int \mathrm{d}^2\boldsymbol{\rho}\, |\boldsymbol{\rho}\rangle \langle\boldsymbol{\rho}|$ to get

$$\psi_{n_f,j_f}(\boldsymbol{k}) = \frac{2\mu}{\hbar^2}\frac{1}{k_f^2 - k^2 \pm i\eta}\int \mathrm{d}^2\boldsymbol{\rho}\, \mathrm{d}z e^{-i(\boldsymbol{k}-\boldsymbol{k_i})\cdot\boldsymbol{\rho}}\chi^*_{n_f}(z)V_{\mathrm{dd},\,j_f}(\boldsymbol{\rho}, z)\chi_0(z).$$

which uses the notation of equation (SI-6) for the potential.

Calculation of $\Psi_{n_f,j_f}(\boldsymbol{\rho})$

In position representation we have

$$\begin{aligned}\Psi_{n_f,j_f}(\boldsymbol{\rho}) &= \frac{1}{(2\pi)^2}\int \mathrm{d}^2\boldsymbol{k} e^{i\boldsymbol{k}\cdot\boldsymbol{\rho}}\psi_{n_f,j_f}(\boldsymbol{k})\\ &= \frac{2\mu}{\hbar^2}\int \mathrm{d}^2\boldsymbol{\rho_1}\underbrace{\frac{1}{(2\pi)^2}\int \mathrm{d}^2\boldsymbol{k}\frac{e^{i\boldsymbol{k}\cdot(\boldsymbol{\rho}-\boldsymbol{\rho_1})}}{k_f^2 - k^2 \pm i\eta}}_{I} e^{i\boldsymbol{k_i}\cdot\boldsymbol{\rho_1}}\int \mathrm{d}z\chi^*_{n_f}(z)V_{\mathrm{dd},\,j_f}(\boldsymbol{\rho_1}, z)\chi_0(z)\end{aligned}$$

Computing the integral I requires a few steps in Mathematica:

$$\begin{aligned}I &= \frac{1}{(2\pi)^2}\int_0^\infty k\,\mathrm{d}k\int_0^{2\pi}\mathrm{d}\theta_k\frac{e^{ik|\boldsymbol{\rho}-\boldsymbol{\rho_1}|\cos\theta_k}}{k_f^2 - k^2 \pm i\eta}\\ &= \frac{1}{(2\pi)^2}\int_0^\infty k\,\mathrm{d}k\frac{2\pi J_0\left(k|\boldsymbol{\rho}-\boldsymbol{\rho_1}|\right)}{k_f^2 - k^2 \pm i\eta}\end{aligned}$$

$$= \frac{1}{(2\pi)^2}\left(-2\pi K_0\left(\sqrt{-k_f^2 \mp i\eta}|\boldsymbol{\rho}-\boldsymbol{\rho_1}|\right)\right)$$
$$= -\frac{i}{4}H_0^{(1)}\left(\sqrt{k_f^2 \pm i\eta}|\boldsymbol{\rho}-\boldsymbol{\rho_1}|\right) \xrightarrow[\eta\to 0]{} -\frac{i}{4}H_0^{(1)}\left(\sqrt{k_f^2}|\boldsymbol{\rho}-\boldsymbol{\rho_1}|\right),$$

with K_0 being the Bessel K function with a complex argument and $H_0^{(1)}$ the Hankel function of the first kind. We only keep the + solution from the square root in the Hankel function to have an outgoing flux and perform the far field expansion $H_0^{(1)}(k_f|\boldsymbol{\rho}-\boldsymbol{\rho}_1|) \simeq \sqrt{\frac{2}{\pi k_f \rho}}e^{ik_f\rho}e^{-ik_f \boldsymbol{u_\rho}\cdot\boldsymbol{\rho}_1}e^{-i\pi/4}$, so by writing $\boldsymbol{k_f} = k_f\boldsymbol{u_\rho}$ we find

$$\Psi_{n_f,j_f}(\boldsymbol{\rho}) = \frac{2\mu}{\hbar^2}\frac{-i}{4}\sqrt{\frac{2}{\pi k_f\rho}}e^{ik_f\rho}e^{-i\pi/4}\int \mathrm{d}^2\boldsymbol{\rho_1}e^{-i(\boldsymbol{k_f}-\boldsymbol{k_i})\cdot\boldsymbol{\rho_1}}$$
$$\int \mathrm{d}z\chi_{n_f}^*(z)V_{\mathrm{dd},\,j_f}(\boldsymbol{\rho_1},z)\chi_0(z).$$

To further simplify we first introduce the Fourier transform $\mathcal{H}_{n_f}$ of the harmonic oscillator wave functions product $\chi_{n_f}^*(z)\chi_0(z)$:

$$\chi_{n_f}^*(z)\chi_0(z) = \frac{1}{2\pi}\int \mathrm{d}q_z e^{iq_z z}\mathcal{H}_{n_f}(q_z) = \frac{1}{2\pi}\int \mathrm{d}q_z e^{-iq_z z}\mathcal{H}_{n_f}(-q_z),$$

and then define $\boldsymbol{q} = \boldsymbol{k_f} - \boldsymbol{k_i} + q_z\boldsymbol{u_z}$ and $\boldsymbol{r_1} = \boldsymbol{\rho_1} + z\boldsymbol{u_z}$ which gives

$$\Psi_{n_f,j_f}(\boldsymbol{\rho}) = \frac{2\mu}{\hbar^2}\frac{-1}{4}\sqrt{\frac{2}{\pi k_f\rho}}e^{ik_f\rho}e^{+i\pi/4}\frac{1}{2\pi}\int \mathrm{d}q_z\mathcal{H}_{n_f}(-q_z)$$
$$\int \mathrm{d}^3\boldsymbol{r_1}e^{-i\boldsymbol{q}\cdot\boldsymbol{r_1}}V_{\mathrm{dd},\,j_f}(\boldsymbol{r_1}).$$

We introduce the Fourier transform of the dipole-dipole interaction

$$\mathcal{V}_{j_f} = \int \mathrm{d}^3\boldsymbol{r_1}e^{-i\boldsymbol{q}\cdot\boldsymbol{r_1}}V_{\mathrm{dd},\,j_f}(\boldsymbol{r_1}),$$

and define the scattering amplitude f such that

$$\Psi_{n_f,j_f}(\boldsymbol{\rho}) = \frac{e^{ik_f\rho}}{\sqrt{\rho}}e^{i\pi/4}f(\boldsymbol{k_f}-\boldsymbol{k_i},n_f,\Delta m).$$

Therefore

$$f(\boldsymbol{k_f}-\boldsymbol{k_i},n_f,\Delta m) = \frac{\mu}{\hbar^2}\frac{-1}{2\sqrt{2}\pi^{3/2}}\frac{1}{\sqrt{k_f}}\int \mathrm{d}q_z\mathcal{H}_{n_f}(-q_z)\mathcal{V}_{\Delta m}(\boldsymbol{k_f}-\boldsymbol{k_i},q_z). \tag{SI-15}$$

The Fourier transform is

$$\mathcal{V}(\boldsymbol{k}, q_z) = \mu_0 (J g_J \mu_B)^2 \Bigg(\left(\bar{k}_z \bar{k}_z - 1\right) |j_0\rangle\langle j_0| + \frac{1}{J^{1/2}} \bar{k}_z \bar{k}_+ |j_1\rangle\langle j_0| + \frac{1}{2J} \bar{k}_+ \bar{k}_+ |j_2\rangle\langle j_0| \Bigg) \quad \text{(SI-16)}$$

with $\bar{k}_+ = \frac{k_x + i k_y}{\sqrt{k^2 + q_z^2}}$ and $\bar{k}_z = \frac{q_z}{\sqrt{k^2 + q_z^2}}$

Symmetrization

We carried all along a term $e^{i\boldsymbol{k_i}\cdot\boldsymbol{\rho_1}}$ which is $\Psi_0^{k_i}(\boldsymbol{\rho_1})$. To symmetrize the bosonic wavefunction we simply change it to $\frac{e^{i\boldsymbol{k_i}\cdot\boldsymbol{\rho_1}} + e^{-i\boldsymbol{k_i}\cdot\boldsymbol{\rho_1}}}{\sqrt{2}}$ which leads to

$$f_S(\boldsymbol{k_f}, \boldsymbol{k_i}, n_f, \Delta m) = \frac{1}{\sqrt{2}} \left(f(\boldsymbol{k_f} - \boldsymbol{k_i}, n_f, \Delta m) + f(\boldsymbol{k_f} + \boldsymbol{k_i}, n_f, \Delta m) \right).$$

Scattering cross section

The flux is defined through the gradient of the wave function $\frac{\hbar}{\mu}\mathrm{Re}\left[\frac{1}{i}\Psi^* \boldsymbol{\nabla}\Psi\right]$. $\boldsymbol{J_i} = \frac{\hbar \boldsymbol{k_i}}{\mu}$ is the incident current of $\Psi_0(\boldsymbol{\rho}) = e^{i\boldsymbol{k_i}\cdot\boldsymbol{\rho}}$. The main contribution to the outgoing wave current comes from its radial part as the other terms in the gradient fall off as $1/\rho^2$ instead of $1/\rho$: $\boldsymbol{J_f} \simeq \frac{\hbar \boldsymbol{k_f}}{\mu} \frac{1}{\rho} |f_S(\boldsymbol{k_f}, \boldsymbol{k_i}, n_f, \Delta m)|^2$. The differential scattering cross section into an angle $\mathrm{d}\theta_f$ is

$$\frac{\partial \sigma}{\partial \theta_f} \mathrm{d}\theta_f = \frac{k_f}{k_i} |f_S(\boldsymbol{k_f}, \boldsymbol{k_i}, n_f, \Delta m)|^2.$$

So the total scattering cross section is

$$\sigma(\boldsymbol{k_i}, n_f, \Delta m) = \frac{k_f}{k_i} \int \mathrm{d}\theta_f |f_S(\boldsymbol{k_f}, \boldsymbol{k_i}, n_f, \Delta m)|^2.$$

$\beta_{2\mathrm{D}}$ *loss coefficient*

The loss coefficient is $\beta = \sigma v = \sigma \frac{\hbar k_i}{\mu}$. It still depends on the direction of $\boldsymbol{k_i}$. It is then averaged in all possible incoming directions: $\beta_{2D}(k_i, n_f, \Delta m) = \frac{1}{2\pi} \int \mathrm{d}\theta_i \beta_{2D}(\boldsymbol{k_i}, n_f, \Delta m)$ to give

$$\beta_{2D}(k_i, n_f, \Delta m) = \frac{\mu}{\hbar^3} \frac{1}{32\pi^4} \int \mathrm{d}\theta_i \int \mathrm{d}\theta_f \Bigg| \int \mathrm{d}q_z \mathcal{H}_{n_f}(-q_z) \left(\mathcal{V}_{\Delta m}(\boldsymbol{k_f} - \boldsymbol{k_i}, q_z) + \mathcal{V}_{\Delta m}(\boldsymbol{k_f} + \boldsymbol{k_i}, q_z) \right) \Bigg|^2.$$

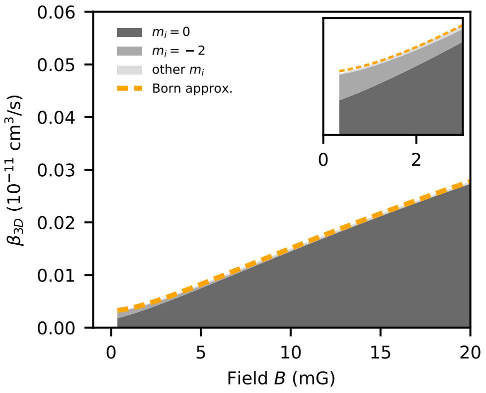

Fig. S1 Comparison between Fermi's golden rule and the Born approximation. The regions shaded in gray are calculated with Fermi's golden rule from equation (SI-9) with free wave functions, including multiple m_i incoming partial waves. The orange line is the Born approximation. The insert zooms in on small magnetic fields.

Comparing Born approximation and Fermi's golden rule

The Born approximation and Fermi's golden rule give the same results once all incoming m_i partial waves are taken into account in the sum in equation (SI-8). This is expected since they are both calculated in first-order perturbation theory. It is not obvious from the final expressions but can be seen in Fig. S1. At high magnetic field, only the $m_i = 0$ channel is contributing since the rate scales as $k_f(k_i/k_f)^{|m_i|}$. However, at low enough magnetic field, the incoming and outgoing momenta are comparable and the other channels become as important, especially the $m_i = -2 \to 0$.

Supplementary Note 4: Pure-2D limit

We mentioned in the main text that the decay rate in the pure-2D case is indefinitely suppressed when the magnetic field increases. The reason is that as the field increases, so does the outgoing momentum, and the relaxation becomes shorter-ranged. The inward region is shielded by the dipolar potential which repels the atoms. We offer here a derivation of the shielded rate in pure-2D. We first derive the un-shielded case. Then we use a known [3] zero-temperature shielded wave function and patch it at long-distance with a free low momentum wave function to compute the shielded rate. This will exhibit the spectacular behavior of a decreasing relaxation rate with an increasing magnetic field. We also show further suppression by going to lower temperature. We have not achieved this regime in our experimental setup, but we believe it is within reach with minor improvements, and think this theoretical treatment can provide insights into the system.

One starts with the equation (SI-10) to write the 2D rate in the case of infinite transverse confinement ($\tilde{\chi}_0(z) = \delta(z)$). The only decay channel is $j_f = 2$ and

$$\beta^{\text{pure-2D}} = \frac{4}{\tilde{k}_i \tilde{k}_f} \frac{E_{\text{dd}}}{\hbar} a_{\text{dd}}^2 \left| \tilde{L} \int_0^{\tilde{L}} d\tilde{\rho} \tilde{\phi}_f(\tilde{\rho}) \tilde{V}_{\text{dd},2}(\tilde{\rho}) \tilde{\phi}_0(\tilde{\rho}) \right|^2 \tag{SI-17}$$

with $\tilde{V}_{\text{dd},2}(\tilde{\rho}) = \left(\frac{-3}{2J}\right) \frac{2}{\rho^3}$.

Free wave functions case

If we ignore the shielding, the wave functions take a simple form $\tilde{\phi}_m(\tilde{\rho}) = \sqrt{\frac{\pi \tilde{k} \tilde{\rho}}{\tilde{L}}} J_m(\tilde{k}\tilde{\rho})$. In the low-temperature limit, the incoming Bessel function goes like $J_0(\tilde{k}_i \tilde{\rho}) \simeq 1$. The integral $\int_0^\infty \mathrm{d}x \frac{J_2(kx)}{x^2} = \frac{k}{3}$ and

$$\beta_{\text{free}}^{\text{pure-2D}} = 4\pi^2 \frac{1}{J^2} \frac{E_{\text{dd}}}{\hbar} a_{\text{dd}}^2 \tilde{k}_f^2. \tag{SI-18}$$

So $\beta_{\text{free}}^{\text{pure-2D}} \propto B$.

Shielded wave functions case

The result is radically different if we take into account the dipolar shielding potential. We will show two effects: the rate decreases both with increasing magnetic field and decreasing temperature. Lower temperature means greater shielding, and higher magnetic field shortens the range of the interaction which increases the shielding as well. To keep the model simple, we will only take the incoming wave function to be shielded since the outgoing one already experiences a centrifugal barrier. In pure-2D, the differential equation for the

incoming wave function follows from equation (6):

$$\left(-\frac{d^2}{d\tilde{\rho}^2}+\frac{-1/4}{\tilde{\rho}^2}+\frac{2}{\tilde{\rho}^3}\right)\tilde{\phi}=\tilde{k}_i^2\tilde{\phi} \tag{SI-19}$$

for which an analytical result is known from [3] but only at zero temperature ($\tilde{k}_i = 0$) and involves modified Bessel functions. At finite temperature and in the absence of the dipolar interaction term, the solution is known with regular Bessel functions. We therefore use the modified Bessel function solution at short-range until $\tilde{\rho}_0$ such that $2/\tilde{\rho}_0^3 = \tilde{k}_i^2$. We then patch this function with the finite temperature free solution with a phase shift δ. The incoming wave function then reads

$$\begin{aligned}\tilde{\phi}_{\text{in}}(\tilde{\rho}) &= \alpha\sqrt{\tilde{\rho}}K_0\left(\sqrt{\frac{8}{\tilde{\rho}}}\right) && \text{for } \rho < \rho_0\\ &= \sqrt{\frac{\pi\tilde{k}\tilde{\rho}}{\tilde{L}}}\left(\cos(\delta)J_0(\tilde{k}\tilde{\rho})-\sin(\delta)Y_0(\tilde{k}\tilde{\rho})\right) && \text{for } \rho \geq \rho_0\end{aligned}$$

where K_0 is the modified Bessel function and J_0 and Y_0 the Bessel functions of the first and second kind. α is a normalization coefficient obtained by equating the wave functions and their derivative at the patching location $\tilde{\rho} = \tilde{\rho}_0$ in a box of length $\tilde{L}$, which gives

$$\alpha = \frac{\left(\cos(\delta)J_0(\tilde{k}_i\tilde{\rho}_0)-\sin(\delta)Y_0(\tilde{k}_i\tilde{\rho}_0)\right)\sqrt{\frac{\pi\tilde{k}_i\tilde{\rho}_0}{\tilde{L}}}}{\sqrt{\tilde{\rho}_0}K_0(\sqrt{\frac{8}{\tilde{\rho}_0}})}$$

with

$$\delta = \arctan\left(\frac{rJ_0(\tilde{k}_i\tilde{\rho}_0)+J_1(\tilde{k}_i\tilde{\rho}_0)}{rY_0(\tilde{k}_i\tilde{\rho}_0)+Y_1(\tilde{k}_i\tilde{\rho}_0)}\right) \tag{SI-20}$$

and

$$r = \frac{1}{\tilde{k}_i}\left(\frac{\tilde{\phi}'(\tilde{\rho}_0)}{\tilde{\phi}(\tilde{\rho}_0)}-\frac{1}{2\tilde{\rho}_0}\right).$$

We now explore independently two limits: low temperature, and high magnetic field.

Low temperature limit

One can find the low temperature behavior of the incoming wave function. We expect the decay rate to be suppressed at low-temperature as the shielding increases. The r coefficient can be rewritten:

$$r = \frac{K_1\left(\sqrt{\frac{8}{\tilde{\rho}_0}}\right)}{K_0\left(\sqrt{\frac{8}{\tilde{\rho}_0}}\right)},$$

which can be used to expand δ through equation (SI-20):

$$\delta \xrightarrow[\tilde{k}_i \to 0]{} \mathrm{Arctan}\left(\frac{\pi}{2\left(3\gamma + \log\left(\tilde{k}_i\right)\right)}\right), \tag{SI-21}$$

to find:

$$\alpha \xrightarrow[\tilde{k}_i \to 0]{} \frac{-2}{\log\left(\tilde{k}_i\right)}\sqrt{\frac{\pi\tilde{k}_i}{\tilde{L}}}.$$

Note that equating equation SI-21 with the form of the phase shift in 2D for a hardcore potential $\tan\delta \simeq \frac{\pi}{2\left(\ln\left(\tilde{k}_i\tilde{a}/2\right)+\gamma\right)}$ allows to recover the universal dipolar scattering result from [3] that $\tilde{a} = 2e^{2\gamma}$. The limit for α gives the following expression for the incoming wave function at low temperature:

$$\tilde{\phi}_{\mathrm{in}}(\tilde{\rho}) = \frac{-2}{\log\left(\tilde{k}_i\right)}\sqrt{\frac{\pi\tilde{k}_i\tilde{\rho}}{\tilde{L}}}K_0\left(\sqrt{\frac{8}{\tilde{\rho}}}\right).$$

This is reflected in the decay rate and $\beta_{\mathrm{shielded}}^{\mathrm{pure\ 2D}} \propto \left(1/\log\left(\tilde{k}_i\right)\right)^2$, which goes to zero at low temperature. This is very different from the free wave function case which had a finite limit even at zero temperature. The increase in the shielding factor with colder temperatures is shown in Fig. 3d.

Moderate magnetic field approximation

Here we derive an approximate analytical formula for the integral in equation (SI-17) with shielded wave functions. Except in the form of complicated Meijer G functions, there is no simple expression for the integral of a Bessel K function multiplied by a Bessel J or Y and the dipolar potential. We have done full numerical calculations (see below). However, we can find an approximate analytical result for moderate magnetic fields by approximating the Bessel functions by ones having known integrals. Fig. S2 explains the different approximations we do to compute the integral. For high-enough magnetic field compared to the temperature, the outgoing Bessel wave function oscillates and has its first zero before the patching point $\tilde{\rho}_0$ (see Fig.S2). This corresponds to $x_2/\tilde{k}_f < \tilde{\rho}_0$ so $x_2\hbar/(a_{\mathrm{dd}}\sqrt{4\mu\mu_B g_J B}) < \tilde{\rho}_0 = (2/\tilde{k}_i^2)^{1/3}$ which gives $B \simeq 0.3\,\mathrm{G}$. We can then cut off the integral at this value as the remaining part is damped by the $1/\tilde{\rho}^3$ potential. This is valid only if the incoming wave function is not increasing too much after this zero, as it would compensate for the decrease due to the potential, which would result in the second lobe of the oscillation contributing more than the first one. The incoming wave function increases exponentially up to a certain distance $\tilde{\rho}_i$ which sets an upper bound on the magnetic field our model tolerates. The upper bound is

$x_2/\tilde{k}_f > \tilde{\rho}_i \simeq 1.40$ which corresponds to $B \simeq 2.3\,\mathrm{G}$. This is on the very conservative side as the model can tolerate fields up to 50 G. Defining $\tilde{D} = x_2/\tilde{k}_f < \tilde{\rho}_0$ with $x_2 \simeq 5.13$ the first zero of $J_2(x)$, the decay rate from equation (SI-17) is

$$\beta^{\text{pure-2D}}_{\text{shielded}} = \frac{4}{\tilde{k}_i\tilde{k}_f}\frac{E_{\text{dd}}}{\hbar}a_{\text{dd}}^2\left|\tilde{L}\int_0^{\tilde{D}} d\tilde{\rho}\sqrt{\frac{\pi\tilde{k}_f\tilde{\rho}}{\tilde{L}}}J_2(\tilde{k}_f\tilde{\rho})\frac{2}{\tilde{\rho}^3}\alpha\sqrt{\tilde{\rho}}K_0\left(\sqrt{\frac{8}{\tilde{\rho}}}\right)\right|^2$$

$$= \frac{36\pi^2}{J^2}\frac{E_{\text{dd}}}{\hbar}a_{\text{dd}}^2\left(\frac{\cos(\delta)J_0(\tilde{k}_i\tilde{\rho}_0) - \sin(\delta)Y_0(\tilde{k}_i\tilde{\rho}_0)}{K_0(\sqrt{\frac{8}{\tilde{\rho}_0}})}\right)^2 \times$$

$$\left|\int_0^{\tilde{D}} d\tilde{\rho}J_2(\tilde{k}_f\tilde{\rho})\tilde{\rho}^{-2}K_0\left(\sqrt{\frac{8}{\tilde{\rho}}}\right)\right|^2.$$

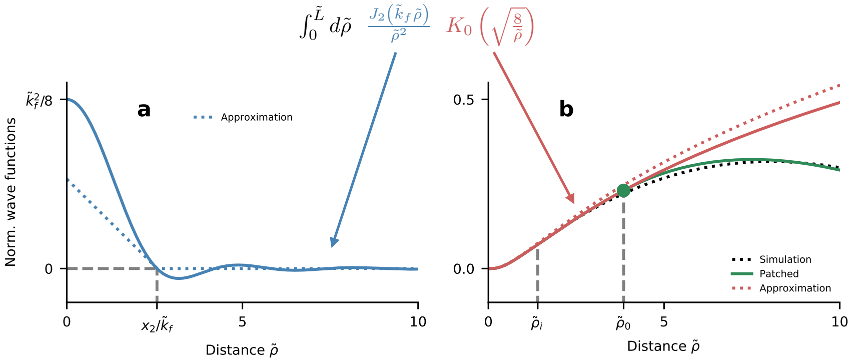

Fig. S2 Illustration of the approximations used to compute the shielded decay rate. (a) The left part of the integrand $J_2(\tilde{k}_f\tilde{\rho})/\tilde{\rho}^2$ corresponding to the outgoing wave function, the potential and the surface element is plotted in solid blue. The outgoing wave function is cut off at the first zero in $x_2/\tilde{k}_f$ and then Taylor expanded at this point inward. (b) The right part of the integrand corresponding to the incoming wave function is plotted in solid red. It is patched in $\tilde{\rho}_0$ to determine the normalization factor, and eventually expanded into a simple exponential function (dashed red). The total patched wave function (red and then green) is compared to the fully simulated wave function (dashed grey). Our model requires $\tilde{\rho}_i < x_2/\tilde{k}_f < \tilde{\rho}_0$.

The patching is only used to determine the normalization factor of $\tilde{\phi}_f$. As we mentioned, we further simplify the functional form of the wave function as illustrated in Fig. S2. We use a Taylor expansion in $\tilde{\rho} = x_2/\tilde{k}_f$ of the Bessel J function

$$\frac{J_2(\tilde{k}_f\tilde{\rho})}{\tilde{\rho}^2} \simeq \tilde{k}_f^3\left(\frac{J_1(x_2) - J_3(x_2)}{2x_2^2}\right)\left(\tilde{\rho} - \frac{x_2}{\tilde{k}_f}\right),$$

and approximate the Bessel K function in $\tilde{\rho} = 0$ by

$$K_0\left(\sqrt{\frac{8}{\tilde{\rho}}}\right) \simeq \exp\left(-\sqrt{\frac{8}{\tilde{\rho}}}\right)\frac{\sqrt{\pi}\tilde{\rho}^{1/4}}{2\cdot 2^{1/4}}$$

The integral

$$\int_0^{\tilde{D}} d\tilde{\rho}\left(1-\frac{\tilde{\rho}}{\tilde{D}}\right)\tilde{\rho}^{1/4}e^{-\sqrt{\frac{8}{\tilde{\rho}}}} = \frac{16}{945}f\left(\tilde{D}\right)$$

with

$$f\left(\tilde{D}\right) = \frac{1}{\tilde{D}}\left[e^{-\sqrt{\frac{8}{\tilde{D}}}}\tilde{D}^{1/4}\left(-256+32\sqrt{2}\tilde{D}^{1/2}+480\tilde{D}-48\sqrt{2}\tilde{D}^{3/2}+21\tilde{D}^2\right)\right.$$
$$\left.-8\cdot 2^{3/4}\sqrt{\pi}\left(-32+63\tilde{D}\right)\operatorname{erfc}\left(\frac{2^{3/4}}{\tilde{D}^{1/4}}\right)\right]$$

gives overall

$$\beta^{\text{pure-2D}}_{\text{shielded}} = \frac{256\pi^3}{99225\sqrt{2}}\left(\frac{J_1(x_2)-J_3(x_2)}{2x_2}\right)^2\times$$
$$\left(\frac{\cos(\delta)J_0(\tilde{k}_i\tilde{\rho}_0)-\sin(\delta)Y_0(\tilde{k}_i\tilde{\rho}_0)}{K_0(\sqrt{\frac{8}{\tilde{\rho}_0}})}\right)^2\frac{1}{J^2}\frac{E_{\text{dd}}}{\hbar}a_{\text{dd}}^2\tilde{k}_f^4 f^2\left(\frac{x_2}{\tilde{k}_f}\right). \quad \text{(SI-22)}$$

The agreement of this analytical expression with the numerical integration is presented in Fig. S3.

High field limit

The complexity of f makes difficult to grasp the behavior of the decay rate β. It is possible to do an expansion at high-magnetic fields of f which greatly simplifies the equation. However this expansion becomes valid only for fields of several thousands of Gauss, which invalidates the initial assumptions that $x_2/\tilde{k}_f$ is larger than the limit $\tilde{\rho}_i$ of the exponentially suppressed region of the incoming wave function. Nonetheless it describes well the overall behavior of the curve, and brings some insight into the suppression of the decay rate. In the large field limit, when setting the numerical and normalization prefactor κ to be:

$$\kappa = \frac{8\pi^3}{105}\left(\frac{J_1(x_2)-J_3(x_2)}{2x_2}\right)^2\left(\frac{\cos(\delta)J_0(\tilde{k}_i\tilde{\rho}_0)-\sin(\delta)Y_0(\tilde{k}_i\tilde{\rho}_0)}{K_0(\sqrt{\frac{8}{\tilde{\rho}_0}})}\right)^2 x_2^{7/4}$$

one obtains

$$\beta_{\text{shielded}}^{\text{pure-2D}} = \kappa \frac{1}{J^2} \frac{E_{\text{dd}}}{\hbar} a_{\text{dd}}^2 \tilde{k}_f^{1/4} \exp\left(-2\sqrt{\frac{8\tilde{k}_f}{x_2}}\right), \tag{SI-23}$$

or $\beta_{\text{shielded}}^{\text{pure-2D}} \propto B^{1/8} \exp\left(-\xi B^{1/4}\right)$, which is a radically different behavior from the free wave function case where the rate is increasing linearly with the magnetic field. Fig. S3 shows this exponential suppression. The suppression factor can be made arbitrarily large. The overall shape of the curve is correctly reproduced by our analytical formulas (SI-22) and (SI-23) even at high fields when $x_2/\tilde{k}_f$ becomes $< \tilde{\rho}_i$. In this case, the decay rate does not get its main contribution from the region defined by the first lobe of the outgoing wave function as the incoming one is not flat but exponentially suppressed in that entire region. Our approximation described in Fig. S2 does not fully capture the precise amplitude of nor the zeros in the decay rate that appear in Fig. S3, however it does give the correct trend of the decay rate. The sharp peaks would blur out with thermal averaging as they strongly depend on the precise position of the nodes of the outgoing wave function with respect to the slow increase of the incoming one. It is also expected that interactions would shift their positions around [1]. Yet, set aside those details, the decay rate should generally decrease with increasing magnetic field due to the shielded character of the incoming wave function and the short-range character of the decay. It is worth noting that other works [4, 5] have studied dipolar shielding of short-ranged losses and found a similar exponential suppression, rising from a WKB approach. The decay factor in the exponential is proportional to $a_{\text{dd}}^{2/5}$ in their case, different from our $\tilde{k}^{1/2} = k^{1/2} a_{\text{dd}}^{1/2}$. The common exponential suppression arises from the same shielding mechanism, however the factors differ as the systems are different: in our case, particles collide at finite momentum in a pure-2D geometry, whereas in the previous works the collision occurs in 3D and at zero temperature. Furthermore, they computed the tunneling rate through the dipolar barrier without assuming any loss mechanism, while we look at the decay rate mediated by the dipolar interaction which extends in our model up to $x_2/\tilde{k}_f$.

Classical turning point

We have explained in the main text how the Franck-Condon principle predicts spin-flips to occur at the classical turning point of the outgoing wave function. The low temperature shielded situation we just presented provides a counterexample where the incoming wave function also has a classical turning point that needs to be taken into account. When the field is sufficiently high, the outgoing wave function oscillates multiple times in the suppressed region of the incoming wave function. Therefore the integrand gets contributions from multiple oscillations, not just the first one.

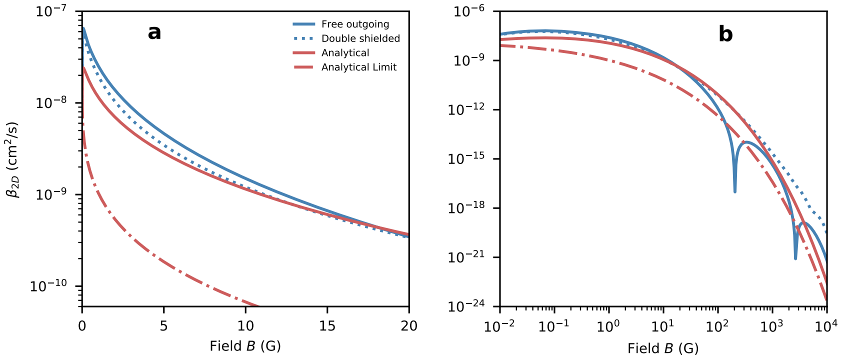

Fig. S3 Dipolar decay rates in two dimensions. Shown are simulated and analytical decay rate coefficients for two magnetic field ranges. Panel (a) is at small magnetic fields and panel (b) shows a broader range. The blue curves represent the rate with simulated wave functions taking into account the dipolar repulsion for both the incoming and outgoing channels (dashed) or only on the incoming one (blue). The red curves present analytical results where the shielding is only accounted in the incoming wave function. The solid line is equation (SI-22) and the dashed one is its high-field limit, equation (SI-23).

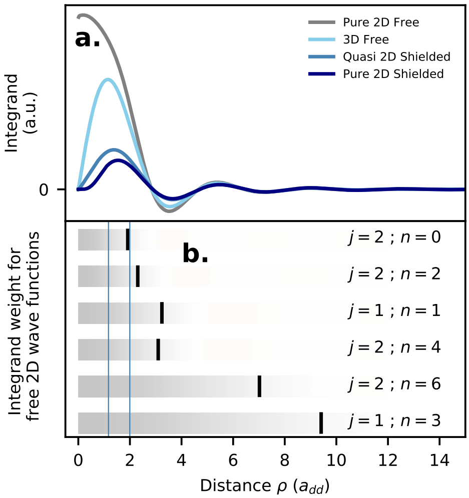

Fig. S4 Integrand of Fermi's golden rule. (a.) The blue curves are the same as in Fig. 1 and come from equation (SI-10). The gray curve, which has been scaled down at short range, represents the fictitious case of a pure-2D geometry using free wave functions. (b.) Amplitude of the same integrand (for free wave functions) shown in grayscale. Multiple n-channels for single $j = 1$ and double $j = 2$ spin flip are presented. The top bar is the same as the gray curve in (a.). The vertical blue lines represent the interval where the dipolar interaction dominates over the other energy terms in equation (6) for the quasi-2D case. The vertical black bars are placed at $\sqrt{12}/k_f$, where k_f is the outgoing momentum.

Supplementary Note 5: Comments on scaling

Integrand behavior

The dipolar relaxation rate in three dimensions has been calculated elsewhere [1, 6, 7]. In reference [1], the authors show that the rate gets its main contribution from the region around the classical turning point of the particles in the exit channel. This can be understood by the fact that the integrand in equation (2) is the product of a flat incoming wave function, a $1/r^3$ potential, a spherical Bessel function that increases as $(k_f r)^2$ and a volume element $4\pi r^2$. The integrand goes like r and increases up to $r \approx 1/k_f$ where the Bessel function starts to oscillate. In two dimensions the story is different, as shown in Fig. S4a. The volume element being $2\pi\rho$, the integrand for free wave functions in pure-2D becomes flat and the contribution to the relaxation rate is homogeneous up to $\sim 1/k_f$. Therefore, there is a large contribution from the inner region, which is classically forbidden.

Going from the fictitious case of free wave functions in 2D to the real quasi-2D case with shielded wave functions has two effects. First, the spin-flip potential gets averaged along z, which reduces the short-range contribution of the integrand. Indeed, in quasi-2D $\langle \frac{\bar{r}_+^2}{r^3} \rangle_{n=0} \propto 1/a_z\rho^2$, which makes the

integrand go to 0 as $\rho \to 0$ (see Fig. S4a, light blue). Then the shielding reduces the short-range amplitude even further (see Fig. S4a, steel blue).

The two vertical blue lines in Fig. S4b indicate the region of space where the dipolar interaction dominates over the centrifugal and the kinetic energy terms. This could also be inferred from Fig. 1a by looking at when the dipolar interaction contribution to the quasi-2D blue curve is bigger than the absolute value of the centrifugal light blue curve and the incoming kinetic energy. The shielding mainly occurs in this region of space and one can only hope to see a reduction of dipolar relaxation from this region inward.

Therefore, the higher the outgoing momentum is, the shorter the range of the interaction is, which increases the shielding factor. This can be seen in Fig. 3c as the shielding factor increases with the magnetic field. Similarly, the excitation of axial motion reduces the final momentum in the radial direction and therefore moves the Franck-Condon point further out. Fig. S4b shows that the integrand contributes far outside the shielded inner region for channels with smaller outgoing radial momentum.

Magnetic field scaling in 3D

We mentioned in the main text that the decay rate for bosons scales as $\sqrt{B}$. This result can be understood with Fermi's golden rule. The outgoing wave function ψ in 3D is an $l = 2$ spherical Bessel function. Its normalization condition in a sphere of radius L gives $\psi \propto k_f/\sqrt{L} j_2(k_f r)$. It therefore rises as $k_f^3 r^2$ for small r before it starts oscillating at $1/k_f$. By integrating the product of the incoming flat wave function, the outgoing one, the volume element $4\pi r^2$ and the potential $1/r^3$ between 0 and $1/k_f$, one gets a matrix element proportional to k_f. As we are considering spherical waves indexed by k_f, we use the one-dimensional density of states $\propto 1/k_f$. This overall gives

$$\Gamma_{\text{3D}} \propto k_f \propto \sqrt{B}.$$

Getting this same result by summing all the contributions from the 2D channels is more complicated as the harmonic oscillator's wave functions play a role, but one can see in Fig. 2b that the free 2D curve eventually meets the 3D one.

Springer Nature 2021 LaTeX template

References

[1] Pasquiou, B., Bismut, G., Beaufils, Q., Crubellier, A., Maréchal, E., Pedri, P., Vernac, L., Gorceix, O., Laburthe-Tolra, B.: Control of dipolar relaxation in external fields. Phys. Rev. A **81**, 042716 (2010)

[2] Du, L., Barral, P., Cantara, M., de Hond, J., Lu, Y.-K., Ketterle, W.: Atomic physics on a 50 nm scale: Realization of a bilayer system of dipolar atoms. arXiv (2023)

[3] Ticknor, C.: Two-dimensional dipolar scattering. Phys. Rev. A **80**, 052702 (2009)

[4] Büchler, H.P., Demler, E., Lukin, M., Micheli, A., Prokof'ev, N., Pupillo, G., Zoller, P.: Strongly correlated 2d quantum phases with cold polar molecules: Controlling the shape of the interaction potential. Phys. Rev. Lett. **98**, 060404 (2007)

[5] Julienne, P.S., Hanna, T.M., Idziaszek, Z.: Universal ultracold collision rates for polar molecules of two alkali-metal atoms. Physical Chemistry Chemical Physics **13**(42), 19114–19124 (2011)

[6] Hensler, S., Werner, J., Griesmaier, A., Schmidt, P., Görlitz, A., Pfau, T., Giovanazzi, S., Rzażewski, K.: Dipolar relaxation in an ultra-cold gas of magnetically trapped chromium atoms. Applied Physics B **77**(8), 765–772 (2003)

[7] Burdick, N.Q., Baumann, K., Tang, Y., Lu, M., Lev, B.L.: Fermionic suppression of dipolar relaxation. Phys. Rev. Lett. **114**, 023201 (2015)
